# Inhibition of the medial amygdala disrupts escalated aggression in lactating female mice after repeated exposure to male intruders

**DOI:** 10.1038/s42003-022-03928-2

**Published:** 2022-09-16

**Authors:** María Abellán-Álvaro, Fernando Martínez-García, Enrique Lanuza, Carmen Agustín-Pavón

**Affiliations:** 1 Unitat Mixta d’Investigació Neuroanatomia Funcional, Departament de Biologia Cel·lular, Funcional i Antropologia Física, Universitat de València, Burjassot, Spain; 2 Unitat Mixta d’Investigació Neuroanatomia Funcional, Unitat Predepartamental de Medicina, Universitat Jaume I de Castelló, Castelló de la Plana, Spain

**Keywords:** Social behaviour, Olfactory system

## Abstract

Virgin female laboratory mice readily express pup care when co-housed with dams and pups. However, pup-sensitized virgins fail to express intruder-directed aggression on a single session of testing. To study whether repeated testing would affect the onset and dynamics of maternal or intruder-directed aggression, we tested dams and their accompanying virgins from postpartum day 4 to 6. Repeated testing led to escalated aggression towards male intruders in dams, but virgins never developed aggression. In dams, inhibition of the medial amygdala using DREADD (designer receptors exclusively activated by designer drugs) vectors carrying the hM4Di receptor blocked the expected increase in maternal aggression on the second testing day. Our data support that the onset of maternal aggression is linked to physiological changes occurring during motherhood, and that medial amygdala, a key centre integrating vomeronasal, olfactory and hormonal information, enables the expression of escalated aggression induced by repeated testing. Future studies selectively targeting specific neuronal populations of the medial amygdala are needed to allow a deeper understanding of the control of experience-dependent aggression increase, a phenomenon leading to the high aggression levels found in violent behaviours.

## Abstract (web summary)

The onset of maternal aggression in mice is dependent on physiological changes that occur during pregnancy and lactation, and the medial amygdala is key in the expression of escalated aggression induced by repeated testing.

## Introduction

Maternal behaviour is one of the most important social behaviours, and its expression requires many genetic and physiological changes in the brain of females^1–3^. These changes enable dams to cope with the new situation and improve the likelihood of offspring survival. Thus, reproductive fitness and maternal behaviour are closely related^1–4^.

One of the most remarkable changes in the behaviour of dams is the onset of maternal aggression^5,6^. In general, rodents exhibit an innate and immediate aggression towards an unfamiliar conspecific placed in the maternal home cage, either in the presence^7,8^ or absence of the pups in the cage during the tests^6,9^. Maternal aggression is in fact a mechanism of defence of the offspring against intruders, and its direct relation to offspring survival endows it with a huge adaptive value. This is particularly true in rodents, where infanticide by non-parental conspecifics is quite common^6,10,11^.

Thus, lactating mothers readily attack intruders, but the intensity of aggression depends on the characteristics of the intruder. In laboratory mice of the CD1 strain, aggression is especially intense against adult males^9,12^. In other lab mice and rats, maternal aggression varies across the postpartum period, gradually decreasing as pups grow old and become more independent^13–15^. Previous results from our lab showed that virgin female mice co-housed with dams and their litters across pregnancy and lactation readily take care of the pups^9^. However, they fail to display aggressive behaviours towards intruder males, at least during their first exposure to them^9,12^. This suggests that intruder-directed aggression is more dependent on hormonal changes occurring during pregnancy and/or lactation than pup care, at least in laboratory mice. In addition, maternal aggression might require a certain degree of motivation to attack as well as a learning process, e.g., the first encounter with an intruder approaching the nest would not result in immediate, effective attacks. In fact, recent results from our lab show that, whereas virgin females co-housed with dams are as quick as dams in classic pup-retrieval tests^9^, they are significantly slower in learning to retrieve pups in a motivated pup-retrieval test in which they need to climb a barrier to access pups^16^. Thus, one could hypothesise a similar situation for intruder-directed aggression, i.e., pup-sensitised virgins co-housed with dams could develop aggressive responses after repeated confrontations with intruders. By contrast, CD1 virgin females that are not co-housed with dams, but only with pups for 2 h per day, needed 3 days to achieve a performance comparable to that of dams in a classical pup-retrieval test^9^ and did not perform any retrieval in the motivated pup-retrieval test^16^. Thus, in the present study, we first characterised how repeated exposure towards an intruder male could affect the onset and features of social and aggressive behaviours of dams and their pair-housed pup-sensitised virgins.

In rodents, most sociosexual behaviours, including maternal aggression, are mainly mediated by chemosensory signals. Rodents possess highly developed olfactory and vomeronasal systems, and chemosignals, pheromones and odours are critical for intraspecies communication^17^. Olfactory cues may be the basis of how dams respond to an intruder’s age, sex, and reproductive status^6,18^. Urine is usually considered the most important source of chemosignals in mice, and urine marking and countermarking is highly displayed by male mice^19^. In particular, a male-specific major urinary protein named *darcin* elicits females’ inherent sexual attraction towards males^20^. However, this same pheromone induces aggressive behaviours in lactating females against an intruder male^12^. The changes in the behavioural responses of females likely involve plastic changes in the sociosexual brain circuits, which could result from a mixture of endocrine agents and sensory inputs (chemosignals, nipple stimulation, etc.) acting during pregnancy, parturition and lactation^6,9,14,18,21,22^.

Protein pheromones such as *darcin* are processed by the vomeronasal system^19^. Within this sensory system, the medial amygdala (MeA) receives direct inputs from AOB^23,24^ and expresses receptors for steroid hormones^25,26^ and for prolactin^22^. Moreover, we recently showed extensive changes in gene expression in the MeA during lactation^3^. The MeA projects to distinct nuclei of the hypothalamus involved in social and defensive responses^27–29^ and plays a central role in the vomeronasal–sensorimotor transformation that leads to specific behavioural responses^30^. Thus, disruptions in MeA activity cause deficits in several social behaviours^31–33^, including maternal care^34^, intermale aggression^35,36^ and maternal aggression^35^. Importantly, a recent study has shown that the MeA is critical in the experience-dependent increase in aggression observed in males^37^. In this framework, we hypothesised that MeA would play a key role in the expression of maternal aggression, and maybe also in the escalated aggression induced by repeated testing of nest defence in postpartum females. To test this hypothesis, we selectively inactivated the MeA by designer receptors exclusively activated by designer drugs (DREADD) in lactating dams during encounters with an intruder male. Our results confirm that pup-sensitised virgins do not become aggressive even after repeated testing, whereas experience results in escalated aggression in dams. In addition, we show that MeA plays a key role in the expression of enhanced maternal aggression.

## Results

### Experiment 1: effect of repeated exposure to a male intruder in intruder-directed aggression in dams and pup-sensitised virgin female mice

**Fig. 1:**
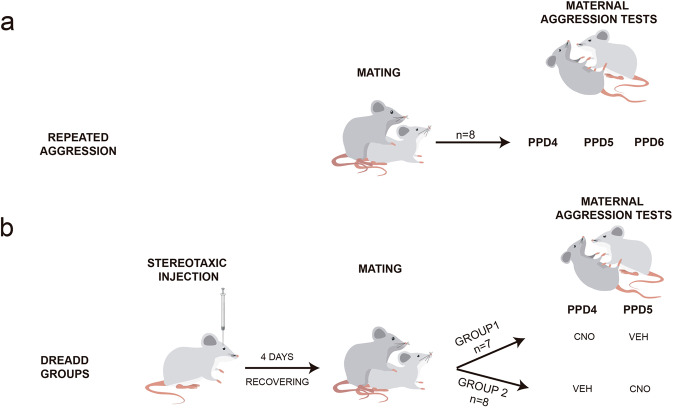
Outline of the protocols followed in the study of maternal aggression behaviour. **a** In Experiment 1, repeated aggression, females were randomly assigned into two groups: dams (*n* = 8) and pup-sensitised females (*n* = 8) (not represented). On postpartum days (PPD) 4–6, both groups of females were exposed to a different intruder male each day. **b** In Experiment 2, effect of DREADD-inhibition of MeA in aggression, females had stereotaxic injections of DREADD-AAV in the MeA. Then, they were mated and randomly assigned to two groups. After parturition, for behavioural testing, we followed an intragroup design. Group 1 received i.p. injection of CNO in PPD4 and Veh in PPD5, and Group 2 received Veh injection in PPD4 and CNO in PPD5. Then, 30 min after i.p. injections, females were exposed to a different male intruder each day. In parallel, we run two control groups of females, without DREADD injection, and the same drug regime, to discard any effect of CNO treatment (Supplementary information). CNO clozapine N-oxide, Veh vehicle.

**Fig. 2:**
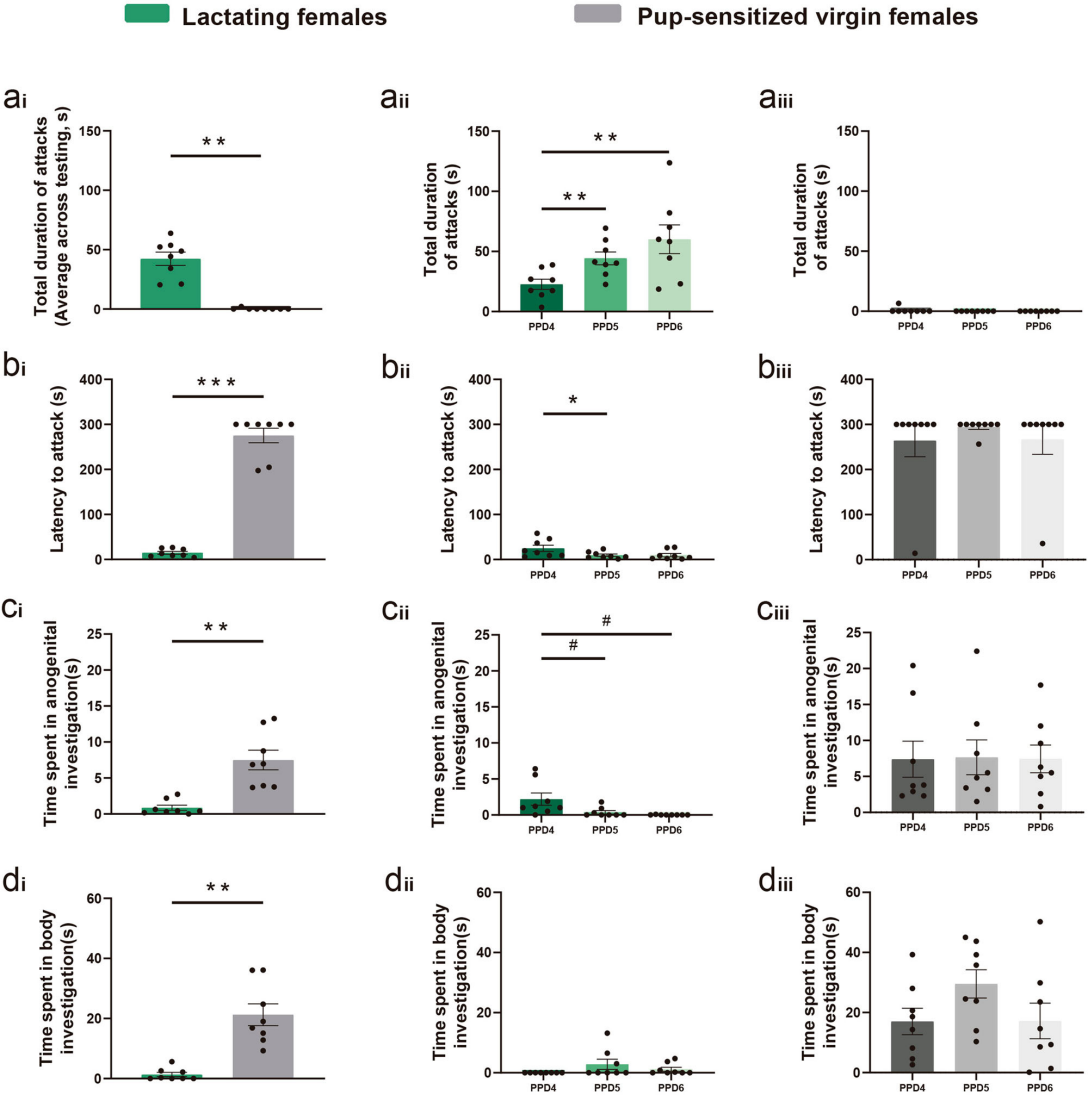
Lactating females displayed increasing levels of maternal aggression across testing days, whereas pup-sensitised females did not develop intruder-directed aggression. **a** Total duration of attacks averaged across testing days was significantly higher in dams than in pup-sensitised virgins. **a’** In lactating females, repeated testing leads to an increase in the total duration of attacks. **a”** Attacks were negligible in pup-sensitised females across testing days. **b** The latency to attack was significantly shorter in lactating females than in pup-sensitised virgin females. **b’** In lactating females, the latency to the first attack was significantly shorter on the second day of testing than on the first. **b”** Since attacks were negligible in pup-sensitised females, latency to attack did not change across testing. **c**, **d** Social investigation was significantly higher in pup-sensitised females than in lactating dams, and did not significantly change across testing days, except for a trend towards decreased anogenital investigation in lactating dams (**c’**). Data are represented as mean ± SEM. **p ≤ 0.01, *p < 0.05, ^#^*p* = 0.058.

First, we compared the overall levels of intruder-directed aggression, latency to attack, anogenital investigation and body approach to males in dams and pup-sensitised virgins. To do so, we calculated for each animal the average duration of each behaviour across the three postpartum days of testing (Fig. 1a). Overall, all dams displayed maternal aggression towards male intruders on all of the testing days (Fig. 2ai, aii, bi, bii), and negligible levels of social investigation (Fig. 2ci, cii, di, dii). By contrast, pup-sensitised females displayed negligible levels of aggression (one subject out of eight attacked on the first day of testing only, whereas another individual displayed one very short bout of attack on the second and third days (Fig. 2aiii, biii), but engaged in anogenital and body approaches towards male intruders (Fig. 2ciii, diii)).

A non-parametric Mann–Whitney test revealed statistically significant differences between both groups of females in all studied behaviours: total duration of attacks (Fig. 2ai), latency to attack (Fig. 2bi), anogenital approaches (Fig. 2ci) and body approaches (Fig. 2di) (*U* = 0; *p* < 0.01; in all the behaviours). Thus, repeated exposure to a male intruder does not induce the onset of intruder-directed aggression in pup-sensitised females.

Next, we analysed whether the levels of aggressive displays changed across testing days in dams. For total duration of attacks, a repeated measures ANOVA showed a significant effect of DAY (*F*_2,6_ = 9.988, *p* = 0.012). Bonferroni post hoc revealed a significant increase in the time that the dams were attacking the male intruder between PPD4 and PPD5 (*p* = 0.009), and between PPD4 and PPD6 (*p* = 0.01) (Fig. 2aii). The repeated measures ANOVA of the log-transformed latency to attack showed a significant effect of DAY (*F*_2,14_ = 4.5, *p* = 0.031). The Bonferroni post hoc analysis revealed significant differences between PPD4 and PPD5 (*p* = 0.042) (Fig. 2bii). Finally, we analysed the structure of maternal aggression across testing days, and we observed significant increases in both the mean duration of individual attacks and in the number of longer attacks (Supplementary Fig. 1). In conclusion, both the duration and the intensity of aggression increased significantly across testing days, with dams displaying significantly more attacks of longer duration the last day of testing as compared to the first day.

Since almost all interactions with the intruder males were aggressive, dams displayed very low levels of exploratory behaviour. In the case of anogenital approaches, this parameter decreased across testing days as aggression increased, as shown by the Friedman test for non-parametric samples (*χ*^2^(2) = 13, *p* = 0.002). The post hoc Wilcoxon signed-rank tests with Bonferroni-adjusted significance level (*α* = 0.017) revealed a trend towards a decrease between PPD4 and the other two days (*p* = 0.058) (Fig. 2cii). This would suggest that, during the first day, dams took a little time to recognise the intruders, whereas in successive testing this time was reduced to almost zero during the last testing session. Concerning dams’ approaches to males’ body, there were no significant differences between the mean ranks of the related groups (*χ*^2^(2) = 4,588, *p* = 0.101) in the Friedman test (Fig. 2dii).

In contrast to dams, pup-sensitised females displayed sociosexual investigation but not maternal aggression towards males. The aggressive behaviour of pup-sensitised females was very low (in fact, tending to 0), and the Friedman test for non-parametric samples did not reveal statistically significant differences between the mean ranks of the related groups (*χ*^2^(2) = 0, *p* = 1) in the total duration of attacks (Fig. 2aiii). Regarding sociosexual investigation, neither anogenital approaches to males (*F*_2.6_ = 0.33, *p* = 0.967) (Fig. 2ciii), nor body approaches (*F*_2.6_ = 3.537, *p* = 0.097) (Fig. 2diii) were significantly different across testing days.

To check whether the behaviour of the females across testing sessions was influenced by the reactions of intruder males, we analysed the time that they spent in initiating an interaction with the females. We found that males rarely approached the dams due to the high rate of attacks and did not significantly change their interaction levels with pup-sensitised females (Supplementary Fig. 2).

### Experiment 2: effect of DREADD-induced inhibition of MeA in maternal aggression

**Fig. 3:**
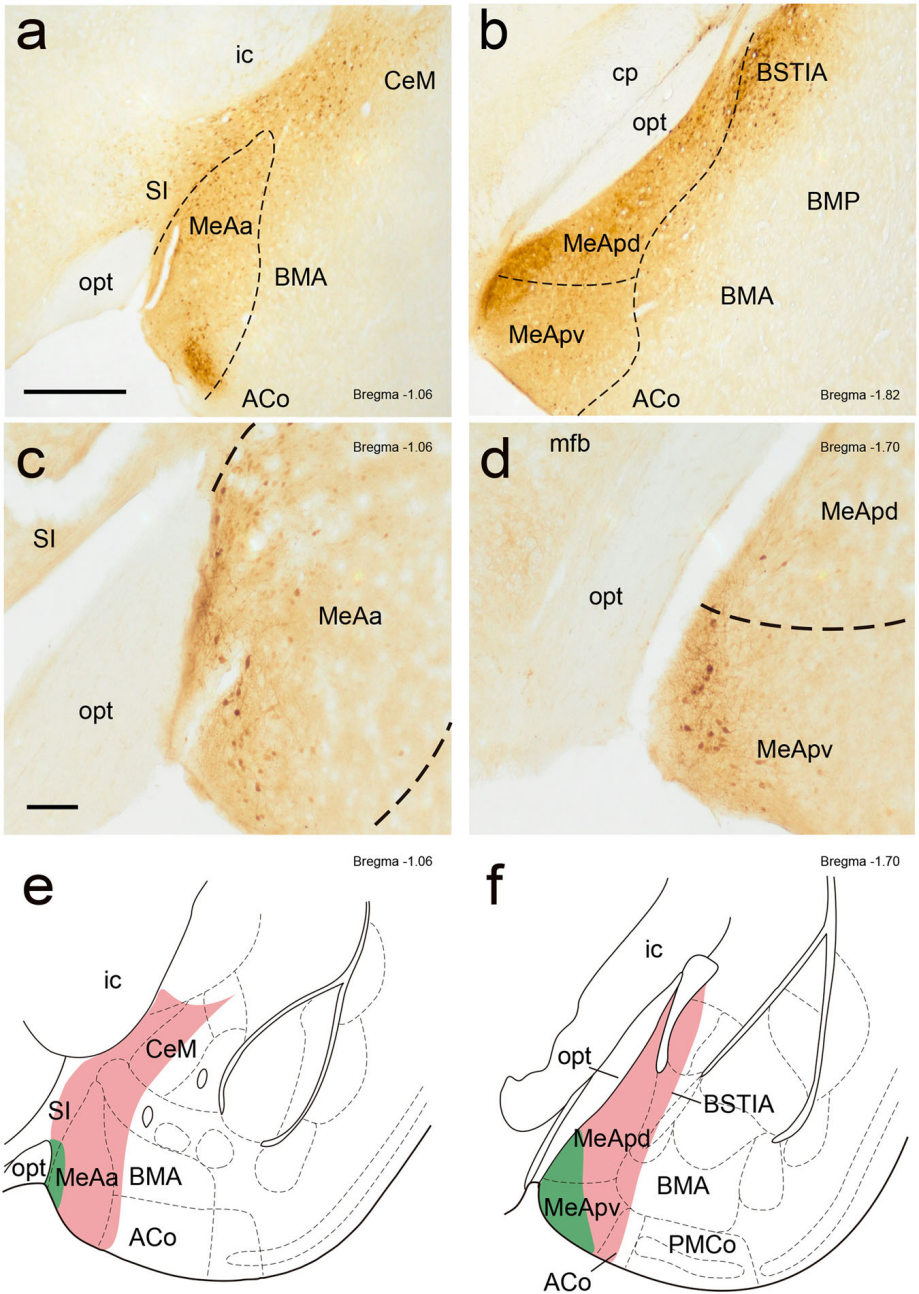
Injection sites in the MeA showing the rostro-caudal extent of the DREADD infections. Photomicrographs showing the biggest (**a**, **b**) and the smallest (**c**, **d**) injection included in the analysis. Scale bar **a**, **b**: 500 µm and **c**, **d**: 100 µm. **e**, **f** Schematic reconstruction of coronal brain sections showing the extent of DREADD infections. Different coloured outlines represent the biggest (light pink) and the smallest (green) injection. The brain drawings are modified from ref. ^61^. ACo anterior cortical amygdaloid nucleus, BMA anterior basomedial amygdaloid nucleus, BSTIA bed nucleus of the stria terminalis intraamygdaloid division, CeM medial part of the central amygdaloid nucleus, ic internal capsule, MeAa anterior medial amygdaloid nucleus, MeApv posteroventral medial amygdaloid nucleus, opt optic tract (opt), MeApd posterodorsal medial amygdaloid nucleus, SI substantia innominate.

This experiment was designed to study the effect of MeA inactivation in maternal aggression (Fig. 1b). Fifteen injections targeted the MeA (Supplementary Table 1). The extent of the injection sites was considered as the area showing an intensely dark extracellular deposit of HRP reaction product and affected mainly the different MeA subnuclei and several adjacent areas in a variable manner (Fig. 3a–f and Supplementary Table 1).

**Fig. 4:**
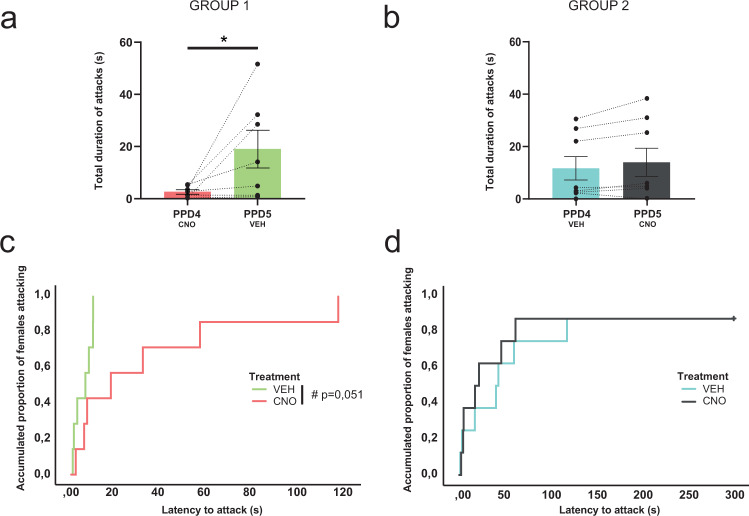
Inhibition of MeA did not block maternal aggression but prevented its increase during the second day of testing. In Group 1, injected with CNO in PPD4 and VEH in PPD5, the ANOVA showed significant differences between PPD4 and PPD5, with an increase in the total duration of aggression during the second session of testing (**a**) and a trend towards a decrease in latency to attack on the second day of testing (**c**). For Group 2, injected with Veh in PPD4 and CNO in PPD5, the ANOVA did not reveal significant differences between studied days in the total amount of time (**b**) or the latency to attack (**d**). Data are represented as mean ± SEM; *p ≤ 0.05, ^#^*p* = 0.051. CNO clozapine N-oxide, Veh vehicle.

In agreement with the results of Experiment 1, except for two of the animals of Group 1, which attacked only on one of the testing days, the rest of the dams displayed aggression towards male intruders on both testing days (Fig. 4). We first analysed the log-transformed data of total duration of attacks by means of a repeated measures ANOVA with the factors DAY (PPD4, PPD5) and TREATMENT (for Group 1, CNO in PPD4 and Veh in PPD5; for Group 2 Veh in PPD4 and CNO in PPD5). We found a significant effect of the factor DAY (*F*_1,13_ = 8,452, *p* = 0.012) and a significant interaction between the factors DAY and TREATMENT (*F*_1,13_ = 6,95, *p* = 0.021) (Fig. 4a, b). The Bonferroni post hoc revealed a significant increase in the total duration of attacks on the second day of testing in the group of dams injected with Veh in PPD5 (*p* = 0.002), but not in dams injected with CNO in PPD5 (Fig. 4a, b). The post hoc failed to reveal significant differences in PPD4 between both groups (*p* = 0.149), suggesting that inactivation of MeA by means of DREADD/CNO did not completely block the innate expression of maternal aggression during the first encounter with the male intruder. However, we cannot discard that this comparison is statistically underpowered to reveal a weak effect, and that the use of a more powerful tool to inhibit the MeA may reveal a potential role of this structure on initial aggression. In any case, the chemogenetic inhibition of the MeA prevented the increase in aggressive responses of dams on the second day of testing.

We then compared the latency to attack between PPD by means of a Kaplan–Meier log-rank test, since some of the animals did not attack, and therefore, we had some censored values. For Group 1, receiving CNO in PPD4 and Veh in PPD5, we found a trend towards a decrease in the latency to attack in PPD5 (*χ*^2^(1) = 3,813, *p* = 0.051, Fig. 4c), in agreement with the increase in aggression during the second session. By contrast, for Group 2, we found no differences between PPD (*χ*^2^(1) = 0,071, *p* = 0.790 (Fig. 4d). Thus, CNO administration prevented both the expected increase in aggression and the expected decrease in the latency to attack.

Since previous data suggested that CNO could have an effect per se^38^, we run additional control groups without DREADD infection. This experiment did not reveal a significant effect of CNO injection on maternal aggression (Supplementary Fig. 3).

## Discussion

In this study, we characterised in depth the behavioural responses displayed by lactating dams and pup-sensitised virgin female mice across 3 days, PPD4 to PPD6, towards male intruders. Our results show that the aggressive responses of dams significantly escalated with repeated testing. Not only the time spent by dams in attacking males was tripled from the first to the third day of testing, but also the duration of the individual attacks was significantly increased and the latency to attack significantly decreased. By contrast, data confirmed that pup-sensitised virgin females do not display intruder-directed aggression, even after repeated exposure to male intruders. Second, we tested the effect of inactivation of MeA in maternal aggression by means of DREADD technology. Our results show that the inactivation of MeA in dams during the first day of testing does not completely abolish maternal aggression. However, MeA inactivation during the second day of testing prevents the expected increase in aggression levels. Thus, the MeA plays a key role in escalated aggression with experience.

Our results show that the aggressive behaviour of dams increases significantly across 3 days of testing, a result similar to evidence in males showing increased aggression with fighting experience^37,39^. Thus, from the first to the third day of testing, dams devoted more time to attacking males, with longer individual attacks and shorter latencies, as well as a decreased time of anogenital investigation. This effect can be due to two non-exclusive possibilities. First, it is possible that repeated testing promotes learning, allowing the females more readily identify the situation and improving the effectiveness of the aggressive responses. Second, the hormonal profiles and/or changing pup features might modulate aggression across PPD. This latter possibility, however, is not very likely in our study, given the narrow period of our testing, from PPD4 to PPD6, during which pups are still absolutely dependent on the mother.

In this context, Svare and Gandelman^13^ tested lactating mice for aggression during six successive pregnancies and lactation periods, on PPD3, PPD7, PPD11, PPD15 and PPD19. They observed that aggression was rarely displayed during pregnancy, but it appeared after pup delivery. The level of aggressiveness was similarly high during PPD3 and PPD7 and decreased as the lactation period advanced^13^. These data showed that the highest levels of aggression are displayed during the initial period of lactation, when pups are still immobile and suckling is very frequent, as opposed to the final period of lactation, where pups start feeding by themselves, suckling is reduced, and hormonal levels are returning to pre-partum levels. The fact that they did not find differences between PPD3 and PPD7 might be due to the interval of 4 days between tests.

Similar to dams, male mice repeatedly exposed to an intruder increased the levels of aggression and decreased the latency to attack as compared to males exposed only once^37,39,40^. In this framework, previous studies have demonstrated that aggression between conspecifics has an important motivational component, and it can be used as reinforcer of instrumental behaviour^41–43^. Thus, it is likely that the motivation to attack of the dams in our study is increasing across testing.

In addition to the likely increase in motivation, there is probably a learning component in maternal aggression. In fact, in the case of sexual behaviour, receptivity, and in particular lordosis, increases with sexual experience^44–47^. In this case, sensory cues from the male need to be integrated with the hormonal status of females to improve female receptivity^33^. This mechanism is likely similar to the one inducing an enhancement of maternal aggression in lactating females. In support of the learning component, the increase in male aggression induced by previous experience is dependent on long-term potentiation (LTP) in the projection from the MeA to the ventromedial hypothalamus (see below)^37^.

Dams can recognise intruders by their chemical signals and other cues, and they can distinguish between familiar males and females and intruders and only display attacks against the intruders^18^. A previous study showed that the critical cue eliciting maternal aggression in CD1 dams is the pheromone *darcin*^12^, a protein found in the urine of gonadally intact males that induces attraction and associative learning in virgin females^20,48^. *Darcin* seems to activate a specific mechanism of associative learning so that instinctive attraction to spend time near this pheromone is extended both to its learned location and to airborne odours associated with the pheromone^20,48^. Thus, the association of the innately aggressive-inducing (for dams) pheromone with other stimuli such as the sight or the smell of the intruder and with the particular context of nest defence testing might induce learning and subsequent expression of the aggressive response.

In contrast to dams, which readily fight an intruder male and significantly increase aggressive behaviour across testing, pup-sensitised females were not aggressive towards males even after repeated exposure. Previously, work from our group showed that CD1 virgins sharing pup care with dams did not attack males in a single intruder-directed aggression test, even though they displayed comparable, or even increased, levels of pup-directed behaviours than dams^9^. Our current results confirm and extend these observations, showing that pup-sensitised females do not develop intruder-directed aggression even after more extensive training. Thus, the experience-induced escalated aggression observed in males^37^ and dams (our study) is not induced in pup-sensitised virgin females. A potential caveat of our work is that the duration of our test (5 min) fails on the short edge of typical testing durations, which vary from 5 to 15 min (reviewed in ref. ^6^). Thus, it might be that pup-sensitised females might need longer latencies to attack. However, we do not think this is the most likely situation. First, even if during the first session of testing females are not aggressive, they might well develop the motivation to attack with experience (as we argued in the Introduction, based on our results of pup-sensitised females able to learn the motivated pup-retrieval task^16^). Second, in contrast to pup care, which can be induced by mere contact with the dam and the pups, there is evidence that intruder-directed aggression needs both the physiological changes occurring during pregnancy and/or lactation and contact with the pups. In fact, hormone-treated virgin females could display intruder-directed aggression provided continuous contact with newborn pups for a longer period (up to 9 days)^49,50^. Interestingly, virgin females developing aggression displayed enlarged nipples, suggesting that both hormonal treatment and suckling stimulation are necessary to induce maternal aggression^49^.

A critical region involved both in the processing of pheromonal information and in maternal aggression is the MeA^51^. As detailed in the Introduction, it receives convergent vomeronasal^23,24^ and hormonal^22,25^ inputs, and projects to areas related to reproductive, defensive and aggressive behaviours, in particular to the BST and VMH^28,29^. In addition, it presents sparse projections to the nucleus accumbens, olfactory tubercle and ventral tegmental area^28^ that could also mediate the enhanced motivation to attack.

From a functional point of view, c-Fos is increased in the MeA in virgin females after exposure to male pheromones^52^, and also in lactating dams exposed to a male intruder^53^. Thus, MeA is a good candidate to be involved in the increase in aggression shown by experienced dams. In fact, the bilateral ablation of MeApd aromatase neurons using a non-reversible genetic technique (cre-dependent Caspase-expressing viral vectors in cre-aromatase animals) resulted in a reduction of aggression events, both in intermale and maternal aggression tests, but not in the total duration of attacks, suggesting that aromatase positive neurons in MeApd play a role in initiating each attack event^35^. These results suggest a common pathway in neural circuits of aggression in males and females, regulated by steroid hormones (progesterone, oestradiol) and activated by stimuli reaching the MeA. The results obtained in our study, showing that Me inactivation results in a reduction of maternal aggression in experienced dams are thus in agreement with Unger et al.^35^.

**Fig. 5:**
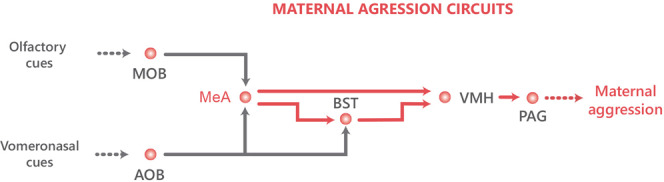
Representation of the social brain network (SBN) nuclei involved in aggression in dam mice related to MeA projections. Projections in red represent circuits with direct experimental evidence for the corresponding behaviour. AOB accessory olfactory bulb, BST bed nucleus of the stria terminalis, MeA medial amygdaloid nucleus, MOB main olfactory bulb, PAG periaqueductal grey, VMH ventromedial hypothalamic nucleus.

Recently, Nordman et al.^37^ showed in male mice that the experienced-induced increase of aggression requires LTP in the projection from the MeA to the ventrolateral part of the VMH (VMHvl) and to the BST. In parallel, also in males, Stagkourakis et al. showed that repeated aggression induces LTP in the VMHvl^40^. The role of MeA is likely related to the integration of chemosensory information from the intruders with the hormonal status of the subject. In males, testosterone plays a role in the aggression-induced LTP in the VMH^40^. In lactating females, we hypothesise that the prolactin and steroid hormone levels influence the activity of the MeA neurons inducing the change from attraction towards male pheromones to aggressive responses. Probably, blocking MeA activity impairs the relay of integrated pheromonal and hormonal information to downstream nuclei that trigger aggressive and motivational behaviours (see nuclei connections in Fig. 5)^54^. The recent finding that maternal aggression is also dependent on the activity of the VMHvl^55^ suggests that the circuit mediating intermale aggression and maternal aggression is the same, and the sexual dimorphism resides in the expression of hormonal receptors in the MeA (and maybe also in the VMH and other MeA targets). In support of this interpretation, the connectivity of the MeA in similar in male^56,57^ and female^28^ mice (and also in male rats)^29^, but the expression of steroid hormone receptors^58^, aromatase^59^, and prolactin^60^ are sexually dimorphic in the MeA.

Similar to the observed role of MeA in an experience-induced increase in maternal aggression, the inhibition of MeA reduces the improvement in receptivity, as measured by lordosis quotient, after repeated exposure to a male^33^. Thus, the inactivation of MeA is likely preventing the integration of pheromonal and hormonal inputs in the sociosexual brain circuits either in mating or in nest-defending contexts.

In summary, our results show that maternal aggressiveness in the first week of lactation increases with repeated testing. In addition, our data confirm that in mice, and at least in our experimental conditions, intruder-directed aggression is not induced in pup-sensitised virgins, even when they are in continuous contact with dams and their litters and after repeated exposure to intruder males. Thus, this behaviour might need the hormonal changes that take place during pregnancy and lactation, which are probably shaping the brain and preparing it for motherhood^22^. The area likely integrating these changes with pheromonal information is the MeA, in which we previously showed significant changes in prolactin-like activity and gene expression across pregnancy and lactation^3,22^. Indeed, chemogenetic inhibition of MeA blocked the experience-induced escalated maternal aggression. Future studies selectively targeting specific neuronal populations of the MeA might allow a deeper understanding of the role of this structure in the control of the onset and expression of aggression.

## Methods

### Experiment 1: effect of repeated exposure to a male intruder in intruder-directed aggression and social investigation in virgin female mice and dams

For this study, we used 16 adult females and 24 male mice (*Mus musculus*) from the CD1 strain (Janvier; Le Genest Saint-Isle, France). Animals were 8–12 weeks old and weighed between 26.8 and 48.5 g at the beginning of the experiments. Adult virgin females were randomly assigned to two groups: dams (*n* = 8) and pup-sensitised females (*n* = 8). Future dams were housed each one with a stud male for 4 days for mating. The day of birth was considered as postpartum day 0 (PPD0). On PPD2, litters were culled to eight pups. Pup-sensitised females were virgin females pair-housed with dams after mating. Consequently, they were continuously exposed to pups and shared pup care with the accompanying dams^9^ (Fig. 1a). Animals were housed in cages with water and food available ad libitum with 12 h light: dark cycle at 22–24 °C. Mice were treated according to the guidelines of the European Union Council Directive of September 22, 2010 (2010/63/UE). All the experimental procedures were approved by the Committee of Ethics on Animal Experimentation of the University of València.

Females were exposed for 5 min from PPD4 to PPD6 to a different intruder male each day in their home cage (Fig. 1a). Before testing, to avoid any vicarious learning, the accompanying female of the experimental subject (a pup-sensitised female in the case of experimental lactating female, or a lactating female in the case of pup-sensitised experimental females) was moved to a clean cage and remained in the houseroom. Then, the home cage was transported to the testing room, and just before testing, pups were carefully removed and placed in a clean container outside of the home cage to avoid any injuries. Behavioural testing was video-recorded and a researcher blind to the experimental groups analysed the number and duration of attacks, latency to attack, anogenital approaches and body approaches, both from female to male and from male to female. Behavioural tests were analysed using the SMART 3.0 video tracking system (Panlab, Barcelona, Spain).

### Experiment 2: effect of DREADD-induced inhibition of MeA in maternal aggression

For this experiment, we used 56 adult females and 82 adult male mice (*Mus musculus*) from CD1 strain (Janvier; Le Genest Saint-Isle, France). Animals were 8–12 weeks old and weighed between 27.8 and 47.9 g at the beginning of the experiments. Females were randomly assigned to four groups depending on the type of injection, in a counterbalanced way. First, two groups of females received a stereotaxic injection of DREADD-AAV, and they were injected with CNO (Clozapine N-oxide) in PPD4 and vehicle on PPD5 (*n* = 17), or vehicle in PPD4 (*n* = 18) and CNO in PPD5 (Fig. 1b).

Animals were housed in cages with water and food available ad libitum with 12 h light: dark cycle at 22–24 °C. Mice were treated according to the guidelines of the European Union Council Directive of September 22, 2010 (2010/63/UE). All the experimental procedures were approved by the Committee of Ethics on Animal Experimentation of the University of València.

Virgin females were anaesthetised with isoflurane (2–2.5%) in oxygen (1–1.3 l/min) (MSS Isoflurane Vaporizer, Medical Supplies and Services, UK) delivered through a mouse anaesthetic mask attached to the stereotaxic apparatus (David Kopf, 963-A, Tujunga CA, USA). For surgery, we administered atropine to prevent cardio-respiratory depression (0.05 mg/kg) and butorphanol as analgesia (5 mg/kg, Turbugesic, Pfizer, New York, USA), both injected subcutaneously. The head was fixed in the stereotaxic apparatus, mice rested on a thermal blanket to keep their body temperature, and were used eye drops (Siccafluid, Thea S.A. Laboratories, Spain) to prevent eye ulceration.

An adeno-associated virus containing the Cre recombinase-independent viral construct AAV5-hSyn-HA-hM4D(Gi)-IRES-mCitrine (B. Roth’s laboratory at the University of North Carolina Vector Core) was used to express the modified human muscarininc inhibitory receptor (hM4Di) and the fluorescent reporter mCitrine in neurons. The AAV was pressure injected bilaterally with a volume of 0.25–0.3 µl (titer of 3.5 × 10^12^ vg/ml) per site at a rate of 0.25–0.3 µl/min. The injections were carried out using glass micropipettes (20–30 µm diameter tips), a 5 µl syringe (Hamilton, Cat# 87900) and a KDS-311 Nano Pump (KD Scientific Inc.). To avoid diffusion of the vector along the pipette track, the micropipette tip was left in place for 10 min after finishing the injection.

Stereotaxic coordinates relative to Bregma were taken from the atlas of the mouse brain^61^ and applied, using a flat skull approach, as follows: A-P: −1.5 to −1.6 mm, M-L (left hemisphere): −2 to −2.2 mm, M-L (right hemisphere): +2 to +2.2 mm and D-V: −4.7 to −5 mm. After the injection, we closed the wound with Histoacryl (Braun, Tuttlingen, Germany) and we applied a second dose of butorphanol. After surgery, we left 2 days of recovery before mating.

After the stereotaxic injections of the viral vector, the females (AAV-injected and control) were caged with a stud male each one for four days to allow mating. Then, pregnant females were singly housed until the end of the experiment. Thus, behavioural testing started 3–4 weeks after infection with the virus. The day of birth was considered as PPD0. As in Experiment 1, litters were culled to eight pups in PPD2.

In PPD4 and PPD5, dams were exposed for 5 min to a different male intruder. Prior to each behavioural test, female subjects were injected intraperitoneally with either CNO (5 mg/kg, Enzo Life Sciences, Farmingdale, NY, USA, diluted in saline (0.9%/PBS) or Vehicle 30 min before the test. Each female was confronted with a male once with and once without CNO treatment, counterbalanced on consecutive days (Fig. 1b). This dose and time of administration have been previously shown to activate DREADD receptors^33,35,62–65^.

All the behavioural tests were video-recorded and analysed by a researcher blind to the experimental conditions. The behavioural responses analysed were number and duration of attacks (bites and chases), anogenital approaches and body approaches, both from female to male and from male to female. All the behavioural tests were analysed using SMART 3.0 video tracking system (Panlab, Barcelona, Spain).

Animals were deeply anaesthetized with an intraperitoneal injection of sodium pentobarbital (100 mg/kg, Eutanax, Laboratories Normon S.A. Madrid, Spain) and perfused transcardially with saline solution (0.9%) followed by 4% paraformaldehyde (diluted in PB 0.1 M, pH 7.6). Following perfusions, brains were removed from the skulls, post fixed for 24 h in the same fixative and cryoprotected in 30% sucrose in PB (0.1 M, pH 7.6) at 4 °C until they sank. We used a freezing microtome to obtain sagittal sections through the olfactory bulbs and frontal sections (both sections of 40 µm) through the rest of the brain. In both cases, sections were collected in four parallel series in 30% sucrose.

One series of each brain was processed for the immunohistochemical detection of mCitrine (the DREADD fluorescent reporter) in free-floating sections. To do so, endogenous peroxidase was inactivated with 1% H_2_O_2_ in Tris-buffered saline (TBS) (0.05 M, pH 7.6) for 30 min at room temperature. Secondly, sections were incubated in blocking solution of TBS-Tx containing 3% normal goat serum (NGS) for 2 h at room temperature. Then, sections were sequentially incubated in: (a) chicken anti-GFP (green fluorescent protein; mCitrine is a variation of GFP) (ABCAM, Cat# ab13970) diluted 1:1000 in TBS-Tx with 2% NGS 72 h at 4 °C; (b) biotinylated goat anti-Chicken IgY (ABCAM, Cat# ab6876) diluted 1:200 in TBS-Tx with 2% NGS for 2 h at room temperature; (c) ABC Elite (Vector, Cat# PK-6100) diluted 1:50 in TBS-Tx for 2 h at room temperature. Finally, the resulting peroxidase labelling was revealed with SIGMA*FAST*^TM^ DAB (Diaminobenzidine) tablets (Sigma-Aldrich Co. LLC, Cat# D4293) dissolved in 10 ml of distilled H_2_O.

Sections were mounted onto gelatinised slides, dehydrated in alcohols, cleared with xylene and coverslipped with Entellan (Merck Millipore).

### Statistics and reproducibility

Statistical analyses were performed using IBM SPSS Statistics 26.0. Firstly, we checked if the data fulfilled the conditions of the ANOVA: normality (Kolmogorov–Smirnov test with Lilliefors’ correction), homoscedasticity (Levene’s test) and sphericity (Mauchly’s test). In the case of non-normality, data were log-transformed (log [*X* + 1]). For Experiment 1, data were analysed using an ANOVA of repeated measures with the factor DAY (PPD4, PPD5 and PPD6) as intra-subject variable. Statistically significant differences (*p* ≤ 0.05) were further explored by means of post hoc pairwise comparisons with Bonferroni’s correction.

When the distribution was not normal even with the logarithmic transformation and the samples were related the non-parametric Friedman’s test was used with the Wilcoxon post hoc signed-rank test when appropriate. In the case of two non-related samples, the non-parametric Mann–Whitney test was used when appropriate.

For Experiment 2, if the data did not follow a normal distribution, it was log-transformed (log [*X* + 1]). Data were analysed using an ANOVA of repeated measures with two factors: DAY (PPD4 and PPD5) as intra-subject factor and TREATMENT (counterbalanced) as between-subjects factor. Statistically significant differences (*p* ≤ 0.05) were further explored by means of post hoc pairwise comparisons with Bonferroni’s correction. In the case of the latency to aggression, some of the animals injected with DREADD did not display aggressive behaviour, and those data were analysed with a Kaplan–Meier log-rank test. The graphs were prepared using GraphPad Prism 8 Software.

### Reporting summary

Further information on research design is available in the Nature Research Reporting Summary linked to this article.

## Supplementary information

Supplementary Information

Description of Additional Supplementary Files

Supplementary Data 1

Reporting Summary

## Data Availability

Source data underlying figures are presented in Supplementary Data 1. Any other microscopic images or quantitative data derived from this study reported in this paper will be shared by the lead contact upon request.
