# Supplementary material for: Inhibition of the medial amygdala disrupts escalated aggression in lactating female mice after repeated exposure to male intruders
Source: Commun Biol. 2022 Sep 16;5:980. doi: 10.1038/s42003-022-03928-2 (PMC9481530; doi:10.1038/s42003-022-03928-2)
Supplement: Supplementary file 1 — Supplementary Information [file 42003_2022_3928_MOESM1_ESM.pdf]

SUPPLEMENTARY INFORMATION

Inhibition of the medial amygdala disrupts escalated aggression in lactating dams after repeated exposure to male intruders

María Abellán-Álvaro[1], Fernando Martínez-García[2], Enrique Lanuza[1], Carmen Agustín-Pavón[1*]

1. Unitat Mixta d'Investigació Neuroanatomia Funcional, Departament de Biologia Cel·lular, Funcional i Antropologia Física, Universitat de València, Burjassot, España

2. Unitat Mixta d'Investigació Neuroanatomia Funcional, Unitat Predepartamental de Medicina, Universitat Jaume I de Castelló, Castelló de la Plana, España.

Supplementary Note 1: Experiment 1. Effect of repeated exposure to a male intruder in maternal aggression in dams and pup-sensitized virgin female mice

We analysed the duration and the structure of aggression by calculating the mean duration of individual attacks and their number. Mean duration of attacks showed significant differences between PPD4 and PPD5 (DAY, $F_{2,6}$=10.074, p=0.012, post-hoc p=0.027) and between PPD4 and PPD6 (post-hoc, p=0.003) (Supplementary Figure 1a). Then, we classified the attacks in three ranges: short (lasting <1s), medium (between 1 and 5 s), and long (> 5 s). The ANOVA revealed a significant effect of the factor RANGE ($F_{2,6}$ = 40.3, p < 0.001), as well as a significant interaction DAY × RANGE ($F_{2,6}$ =7.699, p=0.022). Bonferroni post-hoc revealed significant increase in the number of medium attacks between PPD4 and PPD6 (p=0.02) (Supplementary Figure 1b). Further, whereas only one out of eight tested dams displayed a long attack during PPD4, seven out of eight displayed several long attacks on PPD5 and/or PPD6.

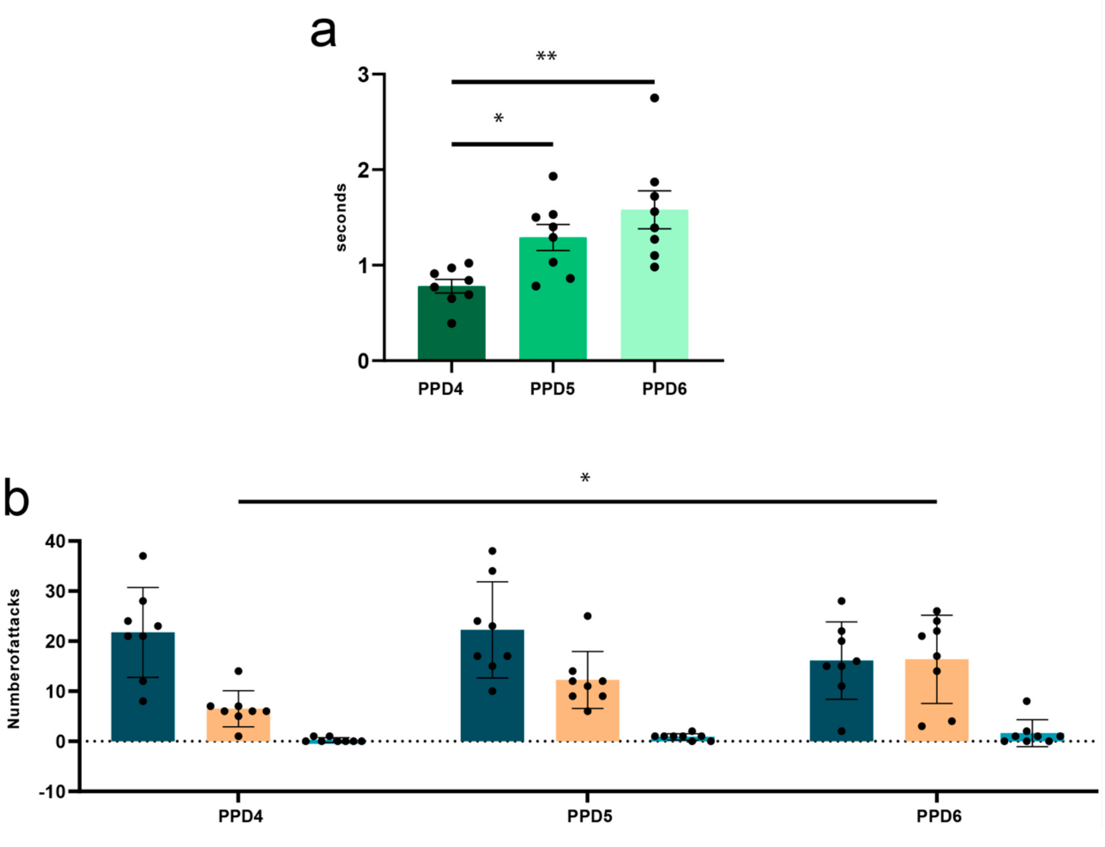

Supplementary Figure 1: Aggression levels of lactating females significantly increased across testing days.

a) The mean duration of individual attacks increased significantly across testing days. b) A histogram of frequencies shows the number of attacks by range of duration (short, 0 to 1 seconds, dark blue; medium, 1 to 5 seconds, orange; long, 5 to 30 seconds, soft blue) from PPD4 to PPD6. The number of medium-length attacks increased significantly from PPD4 to PPD6. Data are represented as mean ±SEM in graph a) and in graph b) as mean ± SD **p≤0.01, *p<0.05.

Males could spend little time displaying ano-genital approaches across testing days ($\chi^2(2)$ =0,154, p=0,926) and could not initiate body approaches to lactating females, since they were extensively attacked (Supplementary Figure 2). In the case of male anogenital approaches to pup-sensitized

females, the repeated measures ANOVA revealed a significant effect of the factor DAY ($F_{2.6}$ =7.485, p=0.023), but Bonferroni post-hoc did not show significant differences between the studied PPD (p>0.05) (Supplementary Figure 2). In the case of males' approaches to the body of pup-sensitized females, the repeated measures ANOVA revealed a significant main effect of the factor DAY ($F_{2.6}$ =0.845, p=0.475) but Bonferroni post-hoc did not show significant differences between the studied PPD (p>0.05) (Supplementary Figure 2). In conclusion, the behaviour of males did not change significantly across testing days, and therefore the behavioural changes observed in females is unlikely to depend on how males behave.

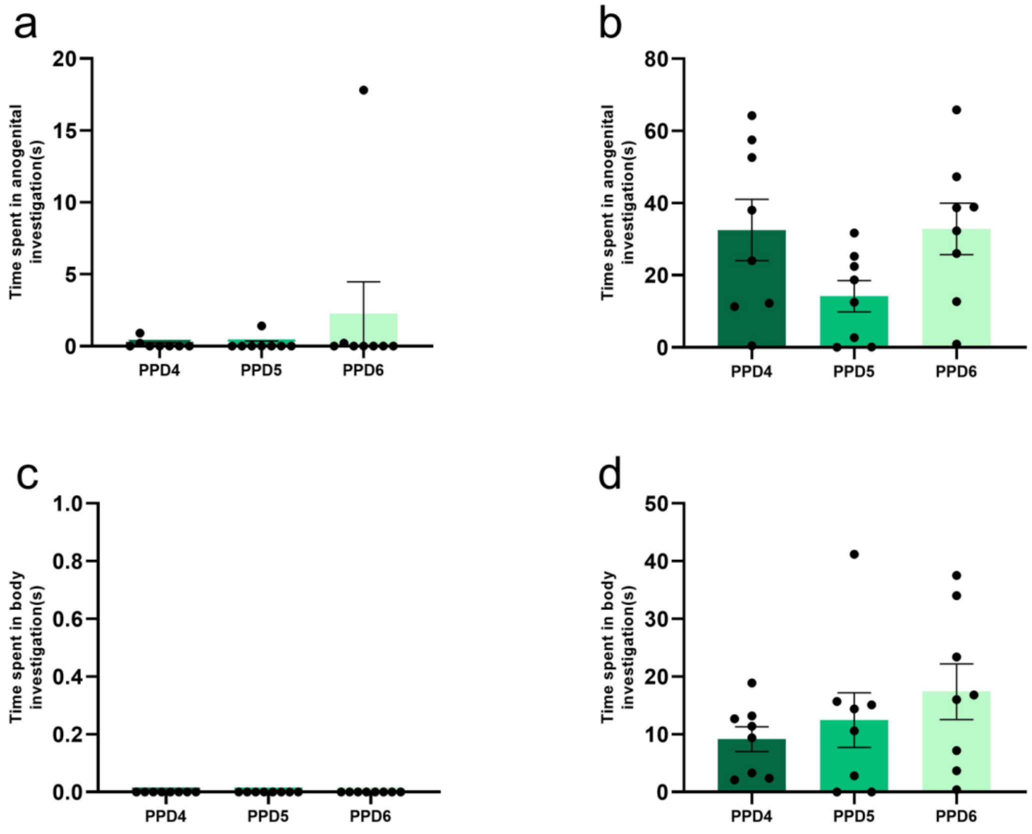

Supplementary Figure 2: Representation of socio-sexual behaviours displayed by males during the testing days.

The behaviour of males toward lactating females was restricted due to the high aggression levels they suffered and did not change across sessions (a and c). However, males showed sociosexual interactions with pup-sensitized virgin females which did not vary across testing days (b and d). Data are represented as mean ± SEM. Postpartum day (PPD).

Supplementary Note 2: Experiment 2. Effect of DREADD-induced inhibition of Me in maternal aggression

Supplementary Table 1: Semiquantitative rating of the injection sites in the Me showing the extent of the DREADD infections in the different nuclei adjacent to the Me. ++++ very dense; + moderate to scarce; 0 not found. Anterior cortical amygdaloid nucleus (ACo), basomedial amygdaloid nucleus (BM), bed nucleus of the stria terminalis intraamygdaloid division (BSTIA), central amygdaloid nucleus (Ce), anterior medial amygdaloid nucleus (MeA), posteroventral medial amygdaloid nucleus (MePV) and posterodorsal medial amygdaloid nucleus (MePD), and supraoptic nucleus (SO).

TREATMENT	SUBJECT CODE	MeA	MePD	MePV	SO	ACo	BM	Ce	BSTIA
GROUP 1 CNO PPD4 VEH PPD5	**1662**	++++	++++	++++	++++	0	0	+	++++
	**1873**	++++	+	++++	++++	0	0	++++	++++
	**1678**	++++	++++	0	0	0	0	0	0
	**1677**	++++	++++	++++	++++	0	0	0	++++
	**1680**	++++	++++	++++	0	0	0	0	++++
	**17105**	++++	++++	++++	++++	++++	++++	+	0
	**1794**	++++	++++	++++	0	0	++++	++++	++++
GROUP 2 VEH PPD4 CNO PPD5	**1665**	++++	++++	++++	+	0	0		++++
	**1667**	++++	++++	++++	++++	+	0	++++	++++
	**1669**	++++	++++	++++	+	0	0	++++	++++
	**1785**	++++	0	0	0	++++	0	0	++++
	**1788**	++++	++++	0	++++	++++	++++	0	0
	**1795**	++++	++++	0	0	++++	0	0	0
	**1709**	++++	++++	++++	0	0	0	0	++++
	**1786**	++++	+	+	++++	++++	0	0	0

To test for the possible effect of CNO or vehicle injections, we added two groups of females without AAV injections, which received CNO injection in PPD4 and Vehicle in PPD5 (n=7) or Vehicle in PPD4 and CNO in PPD5 (n=8). For the total time devoted to aggression, we found a significant main effect of the factor DAY ($F_{1,16}$ = 38,47, p< 0,001), but no significant main effect of TREATMENT ($F_{,16}$ = 0,002, p > 0.6) or DAY X TREATMENT ($F_{,16}$ = 0,017, p > 0.6) (Supplementary Figure 3a-b). Similarly, for the latency to attack we found significant main effect of the factor DAY ($F_{1,16}$ = 15,21, p= 0.001), but no significant main effect of TREATMENT ($F_{1,16}$ = 0,069, p > 0.6) or DAY X TREATMENT interaction ($F_{1,16}$ = 0,003, p > 0.6) (Supplementary Figure 3c-d).

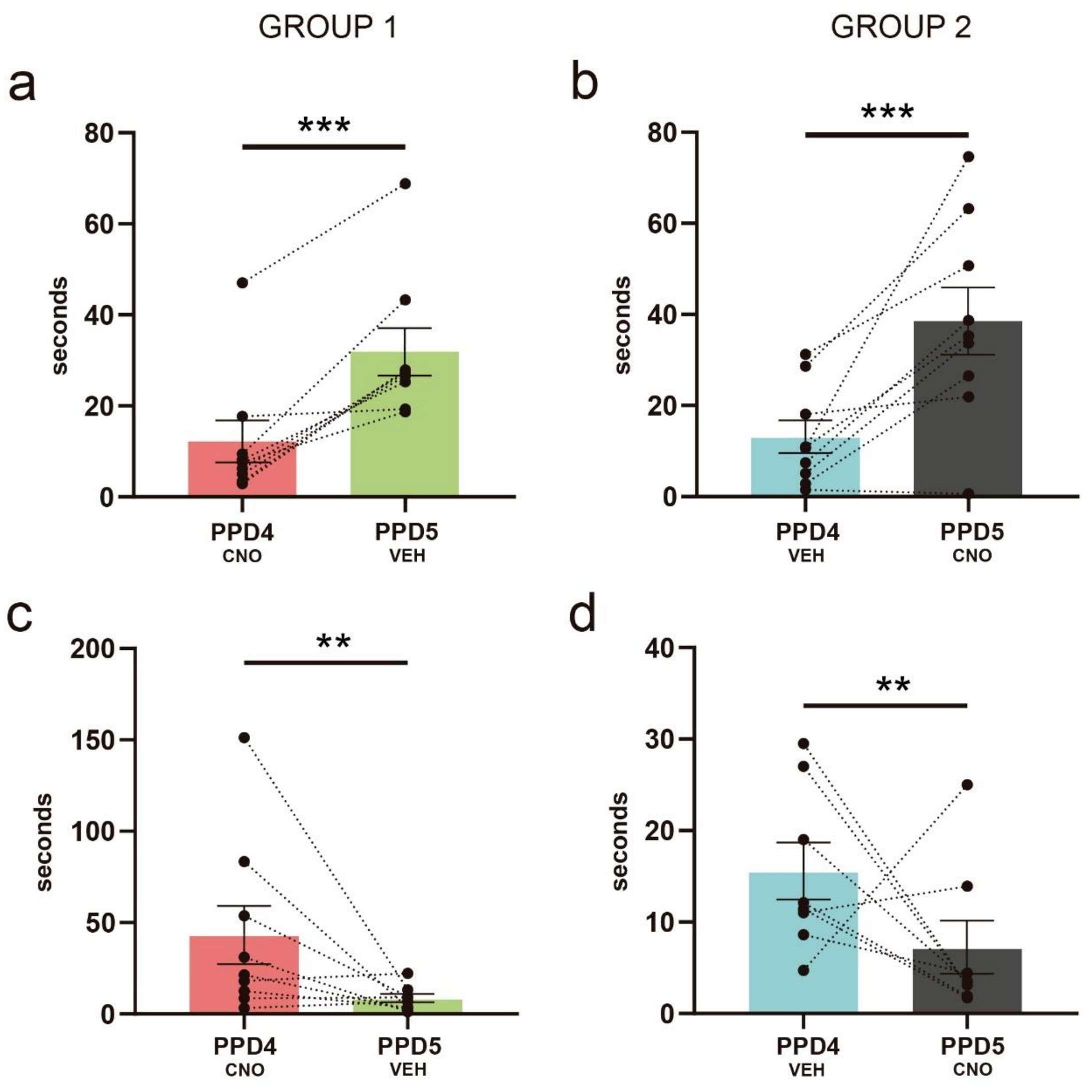

Supplementary Figure 3: CNO injection did not affect maternal aggression levels.

We found a significant increase of maternal aggression between PPD4 and PPD5 irrespective of the intraperitoneal injection of CNO or VEH. Control group 1 with CNO in PPD4 (a); Control group 2 with CNO in PPD5 (b). The latency to attack significantly decrease between tested days regardless of if the CNO injection was in PPD4 (c) or in PPD5 (d). Data are represented as mean ± SEM. ** $p < 0.01$; *** $p < 0.001$.
