# Supplementary material for: Inhibition of the medial amygdala disrupts escalated aggression in lactating female mice after repeated exposure to male intruders
Source: Commun Biol. 2022 Sep 16;5:980. doi: 10.1038/s42003-022-03928-2 (PMC9481530; doi:10.1038/s42003-022-03928-2)
Supplement: Supplementary file 4 — Reporting Summary [file 42003_2022_3928_MOESM4_ESM.pdf]

nature portfolio

Corresponding author(s): Carmen Agustín-Pavón

Last updated by author(s): 29/07/2022

Reporting Summary

Nature Portfolio wishes to improve the reproducibility of the work that we publish. This form provides structure for consistency and transparency in reporting. For further information on Nature Portfolio policies, see our Editorial Policies and the Editorial Policy Checklist.

Statistics

For all statistical analyses, confirm that the following items are present in the figure legend, table legend, main text, or Methods section.

n/a	Confirmed	
☐	☒	The exact sample size (*n*) for each experimental group/condition, given as a discrete number and unit of measurement
☐	☒	A statement on whether measurements were taken from distinct samples or whether the same sample was measured repeatedly
☐	☒	The statistical test(s) used AND whether they are one- or two-sided<br>*Only common tests should be described solely by name; describe more complex techniques in the Methods section.*
☒	☐	A description of all covariates tested
☐	☒	A description of any assumptions or corrections, such as tests of normality and adjustment for multiple comparisons
☐	☒	A full description of the statistical parameters including central tendency (e.g. means) or other basic estimates (e.g. regression coefficient) AND variation (e.g. standard deviation) or associated estimates of uncertainty (e.g. confidence intervals)
☐	☒	For null hypothesis testing, the test statistic (e.g. *F*, *t*, *r*) with confidence intervals, effect sizes, degrees of freedom and *P* value noted<br>*Give P values as exact values whenever suitable.*
☒	☐	For Bayesian analysis, information on the choice of priors and Markov chain Monte Carlo settings
☒	☐	For hierarchical and complex designs, identification of the appropriate level for tests and full reporting of outcomes
☒	☐	Estimates of effect sizes (e.g. Cohen's *d*, Pearson's *r*), indicating how they were calculated

Our web collection on statistics for biologists contains articles on many of the points above.

Software and code

Policy information about availability of computer code

Data collection	NA
Data analysis	NA

For manuscripts utilizing custom algorithms or software that are central to the research but not yet described in published literature, software must be made available to editors and reviewers. We strongly encourage code deposition in a community repository (e.g. GitHub). See the Nature Portfolio guidelines for submitting code & software for further information.

Data

Policy information about availability of data

All manuscripts must include a data availability statement. This statement should provide the following information, where applicable:

- Accession codes, unique identifiers, or web links for publicly available datasets
- A description of any restrictions on data availability
- For clinical datasets or third party data, please ensure that the statement adheres to our policy

Data are available from the corresponding author on reasonable request

Field-specific reporting

Please select the one below that is the best fit for your research. If you are not sure, read the appropriate sections before making your selection.

☒ Life sciences ☐ Behavioural & social sciences ☐ Ecological, evolutionary & environmental sciences

For a reference copy of the document with all sections, see nature.com/documents/nr-reporting-summary-flat.pdf

Life sciences study design

All studies must disclose on these points even when the disclosure is negative.

Sample size	No statistical method was explicitly used to determine the sample size prior to the study. The number of experiments was consistent with the previous research works from the lab. The selected sample size was justified and approved by the Research Ethics and Animal Welfare Committee of the University of Valencia, that granted authorization for the experiments.
Data exclusions	All criteria for data exclusion were decided upon prior to data collection. In the DREADD-behavioral experiments, experimental subjects with off-target placement of stereotaxic injections were excluded from our analyses.
Replication	The same experimental conditions were guaranteed in each of the procedures. We observed similar results which satisfied the same statistical criteria across experiments. Dispersion of data indicated acceptable replication; we have plotted independent data points as a manner to show the consistency of the data.
Randomization	Experimental subjects were randomly assigned to the different experimental groups
Blinding	The experimenters who measured the behaviour of experimental animals were blind to the condition of the animal.

Reporting for specific materials, systems and methods

We require information from authors about some types of materials, experimental systems and methods used in many studies. Here, indicate whether each material, system or method listed is relevant to your study. If you are not sure if a list item applies to your research, read the appropriate section before selecting a response.

Materials & experimental systems

n/a	Involved in the study	
☐	☒	Antibodies
☒	☐	Eukaryotic cell lines
☒	☐	Palaeontology and archaeology
☐	☒	Animals and other organisms
☒	☐	Human research participants
☒	☐	Clinical data
☒	☐	Dual use research of concern

Methods

n/a	Involved in the study	
☒	☐	ChIP-seq
☒	☐	Flow cytometry
☒	☐	MRI-based neuroimaging

Antibodies

Antibodies used	Chicken anti-GFP, ABCAM, Cat# ab13970<br>Biotinylated goat anti-Chicken IgY, ABCAM, Cat# ab6876
Validation	All antibodies used in this study are commercially available and validated by the manufacturers.<br>https://www.abcam.com/gfp-antibody-ab13970.html<br>https://www.abcam.com/goat-chicken-igy-hl-biotin-ab6876.html

Animals and other organisms

Policy information about studies involving animals; ARRIVE guidelines recommended for reporting animal research

Laboratory animals	Subjects were adult CD1 female (n=56) and male mice (n=82) (8-12 weeks old, 30 – 50 g; animal facility of the Central Unit of Research, University of Valencia, Valencia, Spain). Mice were housed in cages on a 12 h light/dark cycle with constant ambient temperature (22 ± 1°C) and humidity, and water and food available ad libitum.
Wild animals	The study did not involve wild animals.
Field-collected samples	The study did not involve samples collected from the field

Ethics oversight	All experimental procedures were approved by the Research Ethics and Animal Welfare Committee of the University of Valencia, in accordance with the European Communities Council Directive (2010/63/UE) on the protection of animals used for scientific purposes.

Note that full information on the approval of the study protocol must also be provided in the manuscript.
